# PEG-assisted reconstruction of the cervical spinal cord in rats: effects on motor conduction at 1 h

**DOI:** 10.1038/sc.2016.72

**Published:** 2016-05-24

**Authors:** C-Y Kim

**Affiliations:** 1 Department of Bioengineering, College of Life Science, CHA University, Seoul, Korea; 2 Department of Laboratory Animal Medicine, College of Veterinary Medicine, Seoul National University, Seoul, Korea

In 1970, White *et al.*^1, 2^ carried out the first cephalosomatic anastomosis (CSA) in primates. However, the experimental animal was left paralyzed because of the inability to reconnect the spinal cord. In 2013, Canavero^3, 4^ made the case for a full human CSA (HEAVEN) in which the atraumatically severed cords of both a brain-dead body donor and a recipient would be reconnected via a newly developed spinal fusion protocol (GEMINI) relying on a special class of substances called fusogens/sealants. In 2014, on the back of Canavero proposal,^3, 4^ a Chinese group reported on the first step toward a full CSA in rats.^5^ This study represents an initial experiment in rats, in which the cervical cord has been sharply transected and PEG applied. In particular, this study describes the preliminary findings of initial motor conduction recovery as assessed acutely (1 h) after sharp severance in view of more extensive trials in the future.

All studies were performed according to animal ethics committees guidelines enacted at the CHA University. Female Sprague–Dawley rats (250–280 g) were anesthetized using zoletil and xylazine (3:1 ratio, 1 ml kg^−1^). Cervical laminectomy was performed using the modified methods of spinal cord injury.^6^ Briefly, the muscles overlying the vertebral column are reflected exposing the vertebral column C4–6 and the C5 spine segment backward is carefully removed. After gently raising the cervical cord with hook, severance was performed with surgical sharp blades #11. Arterial damage including the anterior spinal artery led to bleeding, which, however, could be controlled with cotton swabs and gauzes.

The experimental group (*n*=7) was treated with polyethylene glycol (PEG, MW 400) directly applied on the cervical cord and also dripped on the blades before severance for fast polymer absorption. PEG was selected as the fusogen because of its capacity to repair neuronal membranes after spinal cord injury.^4^ The control group (*n*=7) was treated with phosphate-buffered saline. Dextrose 5% solution was administered daily via intraperitoneal injection, and bladders were catheterized with 24-gauge angiocatheters. Three randomly selected rats per group were analyzed for electrophysiology 1 h after surgery. The remaining 4 rats were utilized for simple behavioral response testing at 1 week and survival evaluation up to 2 weeks.

Immediately after the operation, breathing and heartbeat were maintained spontaneously without special problems; quadriplegia was observed. Importantly, all of the groups receiving the operation survived at least 2 weeks with intensive care, including fluid therapy. After 2 weeks, euthanasia was performed.

To assess electrophysiological conduction of the spinal motor pathway, rats were placed on a stereotaxic device with prone and slightly chin-up position using a tooth bar. Motor-evoked potentials (MEPs) were recorded using a bipolar disk electrode in the sciatic nerve after stimulation (6 mA, 0.1 ms duration) of the hindlimb area of the sensorimotor cortex (Figure 1a). MEP waveforms of normal (sham-operated) animals showed a positive negative deflection pattern (Figure 1b) but did not show a similar pattern in both control and PEG groups.

The recorded amplitude corresponded to the magnitude of the positive-phase peak. Figure 1c shows the average MEP amplitudes recorded following surgery. In controls, MEP amplitude averaged 81.1±12.4 mV at 1 h after surgery, whereas amplitudes in PEG-treated animals were significantly increased compared with the control group (156.7±7.6 mV; *P*=0.0066). Intriguingly, the control group also evinced some transmission following simple apposition of the stumps. After a week, behavioral responses to light stimulation on vibrissae were assessed. Interestingly, both the control and PEG groups showed forefoot movement and occasional spontaneous rear foot movement (Figures 2a and b). Similar with these results, numerous other studies confirm the existence of spontaneous plasticity after spinal cord injury,^6^ and I also suppose that some electrophysiological conduction and behavior response were elected by improved spontaneous recovery with sharp severance because the adjacent stumps suffer less damage.

In this paper, PEG applied to juxtaposed cervical cord stumps can lead to initial neurophysiological motor conduction after only 1 h of a sharp severance. This is a key observation bolstering the proposed HEAVEN/GEMINI spinal cord fusion protocol.^3, 4^

A literature review bears out that the stumps of sharply severed spinal cords can actually be rejoined with functional gains. In 2014, a Polish team^7^ reported on a 38-year-old man with traumatic transection of the spinal cord at T9. Almost 2 years later, one of the patient's olfactory bulbs was removed and used to derive a culture containing olfactory ensheathing cells and olfactory nerve fibroblasts. Following resection of the glial scar, the cultured cells were transplanted into the spinal cord stumps above and below the injury and the 8-mm gap bridged by four strips of autologous sural nerve. The patient underwent an intense pre and postoperative neurorehabilitation program. The patient improved from American Spinal Injury Association (ASIA) A to ASIA C. There was improved trunk stability, partial recovery of the voluntary movements of the lower extremities and an increase in the muscle mass in the left thigh, as well as partial recovery of superficial and deep sensation. There was also some indication of improved visceral sensation and improved vascular autoregulation in the left lower limb. The pattern of recovery suggests functional regeneration of both efferent and afferent long-distance fibers. Imaging confirmed that the grafts had bridged the left side of the spinal cord, where the majority of the nerve grafts were implanted, and neurophysiological examinations confirmed the restitution of the integrity of the corticospinal tracts and the voluntary character of recorded muscle contractions.

The technique employed by the above groups is particularly traumatizing, requiring either abdominal or cranial surgeries with all the attendant risks. Research published since the 1980s strongly suggests that severed axons can be reconstituted by applying special substances that have the power to literally refuse damaged cell membranes: PEG and chitosan.^3, 4^ Recently, after complete chronic transection of the dorsal cord in rats, a PEG-bridge allowed long-distance axon regeneration through the grafted area and for, at least, 1cm beyond the lesion/graft border.^8^ As revealed by electron microscopy, bundles of regenerating axons within the matrix area received myelin ensheathment from Schwann cells. The beneficial effects of PEG-implantation into the resection-cavity were accompanied by long-lasting significant locomotor improvement over a period of 8 months. Following complete spinal re-transection at the rostral border of the PEG-graft, the locomotor recovery was aborted, suggesting a functional role of regenerated axons in the initial locomotor improvement. Thus, scar resection and subsequent implantation of PEG into the generated cavity leads to tissue recovery, axon regeneration, myelination and functional improvement that have not been achieved before in severe chronic SCI.

In this paper, the severance has been effected cervically, as would happen in the context of a full head-body transference.^3, 4^ Being able to observe neurophysiological transmission after a mere hour bolsters the case for full cervical cord fusion. Although these are preliminary results that need to be confirmed by more extensive studies, yet, it appears that immediately re-apposing the stumps of a severed spinal cord can start a process leading to neurophysiological recovery.

## Figures and Tables

**Figure 1:**
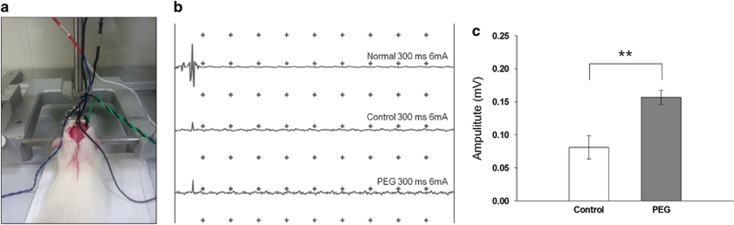
Electrophysiology analysis motor-evoked potential (MEP) measurement (**a**). MEP graphs of normal, control and PEG group (**b**). The average amplitude of control and PEG group after surgery (**c**). Data represent the mean±s.d. of three rats in each group. **P<0.01.

**Figure 2:**
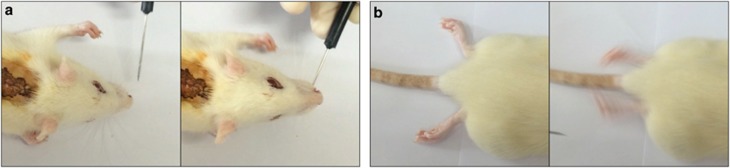
Forefoot behavioral response to vibrissa stimulation (**a**). Spontaneous rear foot movement (**b**).

## References

[bib1] White RJ, Wolin LR, Massopust LC Jr. Taslitz N, Verdura J. Cephalic exchange transplantation in the monkey. Surgery 1971; 70: 135–139. 4997130

[bib2] White RJ, Wolin LR, Massopust LC Jr. Taslitz N, Verdura J. Primate cephalic transplantation: neurogenic separation, vascular association. Transplant Proc 1971; 3: 602–604. 4999463

[bib3] Canavero S. HEAVEN: The head anastomosis venture Project outline for the first human head transplantation with spinal linkage (GEMINI). Surg Neurol Int 2013; 4: S335–S342. 24244881 10.4103/2152-7806.113444 PMC3821155

[bib4] Canavero S. The "Gemini" spinal cord fusion protocol: reloaded. Surg Neurol Int 2015; 6: 18. 25709855 10.4103/2152-7806.150674 PMC4322377

[bib5] Ren XP, Song Y, Ye YJ, Li PW, Han KC, Shen ZL et al. Allogeneic head and body reconstruction: mouse model. CNS Neurosci Ther 2014; 20: 1056–1060. 25367718 10.1111/cns.12341 PMC6493160

[bib6] Lukovic D, Moreno-Manzano V, Lopez-Mocholi E, Rodriguez-Jimenez FJ, Jendelova P, Sykova E et al. Complete rat spinal cord transection as a faithful model of spinal cord injury for translational cell transplantation. Sci Rep 2015; 5: 9640. 25860664 10.1038/srep09640 PMC5381701

[bib7] Tabakow P, Raisman G, Fortuna W, Czyz M, Huber J, Li D et al. Functional regeneration of supraspinal connections in a patient with transected spinal cord following transplantation of bulbar olfactory ensheathing cells with peripheral nerve bridging. Cell Transplant 2014; 23: 1631–1655. 25338642 10.3727/096368914X685131

[bib8] Estrada V, Brazda N, Schmitz C, Heller S, Blazyca H, Martini R et al. Long-lasting significant functional improvement in chronic severe spinal cord injury following scar resection and polyethylene glycol implantation. Neurobiol Dis 2014; 67: 165–179. 24713436 10.1016/j.nbd.2014.03.018

